# Enhanced Humoral and Cellular Immune Responses Elicited by Adenoviral Delivery of SARS-CoV-2 Receptor-Binding Motif Fused to Human Fc

**DOI:** 10.3390/vaccines12111247

**Published:** 2024-11-01

**Authors:** Yea-Jin Lee, Maheswaran Easwaran, Yong-Sam Jung, Yingjuan Qian, Hyun-Jin Shin

**Affiliations:** 1 Laboratory of Infectious Disease, College of Veterinary Medicine, Research Institute of Veterinary Medicine, Chungnam National University, Yuseong-gu, Daejeon 34134, Republic of Korea; yjk274516@naver.com; 2 Department of Research Analytics, Saveetha Dental College and Hospitals, Saveetha Institute of Medical and Technical Sciences, Saveetha University, Chennai 600077, Tamil Nadu, India; maheswaran.easwaran@gmail.com; 3 MOE Joint International Research Laboratory of Animal Health and Food Safety, Jiangsu Foreign Expert Workshop, College of Veterinary Medicine, Nanjing Agricultural University, Nanjing 210095, China; ysjung@njau.edu.cn (Y.-S.J.); yqian@njau.edu.cn (Y.Q.)

**Keywords:** SARS-CoV-2, receptor binding motif, human Fc, ELISPOT assay, enhance immune responses

## Abstract

**Background/Objectives**: The receptor binding motif (RBM) of the SARS-CoV-2 spike protein is critical for viral entry into host cells. Development of a vaccine targeting this region is a promising strategy for COVID-19 prevention. To enhance the immunogenicity of SARS-CoV-2 vaccines, we developed an adenoviral vector expressing the RBM from the SARS-CoV-2 spike protein that fused to the human Fc (hFc) domain. **Methods**: The recombinant RBM_hFc fusion protein was successfully cloned into the pacAd5CMV-N-pA (pAd5) vector and expressed in HEK293 cells as a ~40 kDa protein. A recombinant adenovirus encoding RBM_hFc was subsequently generated and confirmed by cytopathic effect assay. **Results**: Western blot analysis verified the expression of RBM_hFc in the adenovirus (AdV). ELISA assays, validated for IgG detection, demonstrated a twofold increase in IgG antibody levels (M–1.090 at 450 nm; SD—±0.326; and 95% CI—0.250 [0.839 to 1.340]) in sera from BALB/c mice immunized with Ad/RBM_hFc, compared to the negative control group. Result suggests a robust humoral immune response induced by the Ad/RBM_hFc vaccine. Moreover, ELISpot assays demonstrated a tenfold increase in IFN-γ -producing cells (M—440 spot-forming cells; SD—±124.976; and 95% CI—75.522 [364.478 to 515.522]) in mice immunized with AdV/RBM_hFc compared to the negative control group. Result proved that AdV/RBM_hFc-stimulated a robust cellular immune response in animal model. **Conclusions**: Our findings indicate that the RBM_hFc fusion protein enhances both humoral and cellular immune responses. These results suggest the potential of adenoviral vectors carrying RBM_hFc as vaccine candidates. However, comprehensive evaluation of the protective efficacy of these adenoviral vectors will necessitate rigorous experimental studies.

## 1. Introduction

Coronavirus (CoV) is a group of RNA viruses that can cause disease in mammals, particularly respiratory infections such as MERS-CoV, SARS-CoV-1, and SARS-CoV-2 [1]. The SARS-CoV-2 exhibits significantly higher binding affinity (10 to 20-fold) for the human angiotensin-converting enzyme 2 (ACE2) receptor compared to SARS-CoV-1 [2], which leads to efficient cell entry mechanism. In addition, SARS-CoV-2 spreads through saliva droplets and patients have been diagnosed with common symptoms such as cough (76%), fatigue (44%), and fever (98%), as well as atypical symptoms such as headache (8%), diarrhea (3%), and hemoptysis (5%) [3,4]. More specific symptoms such as anosmia, ageusia, and dyspnea were also strongly associated with SARS-CoV-2 [5]. These symptoms highlight the potential for SARS-CoV-2 to cause a severe respiratory disease with a high mortality rate [6]. As of July 2023, the World Health Organization (WHO) reported over 767 million confirmed cases, and 6.9 million deaths associated with SARS-CoV-2 worldwide [7]. The extracellular membrane encloses the single-stranded positive-sense RNA genome of SARS-CoV-2. It is composed of several structural proteins, including the envelope proteins (E), the spike proteins (S), the membrane proteins (M), and the nucleocapsid (N) [8]. In particular, the S protein is crucial for SARS-CoV infection of human lung cells. It consists of two subunits, S1 and S2, and is located on the surface of the virion [9]. The S1 subunit contains the receptor-binding domain (RBD) and recognizes and interacts with the angiotensin-converting enzyme 2 (ACE2) receptor, enabling viral attachment, while the S2 subunit promotes viral–cell membrane fusion through a helical bundle structure [10]. Studies on the receptor-binding motif (RBM) are an emerging area for vaccine development and control of infection mortality rates.

SARS-CoV-2 vaccines target the S-glycoprotein, which helps to neutralize the infection and trigger an immune response [11]. In general, the spike region comprises the subunits S1 (1 to 685 aa) and S2 (686 to 1273 aa). S1 consists of the RBD and an N-terminal domain, while S2 consists of a fusion peptide, a cytoplasmic domain, a transmembrane domain, heptapeptide domain 1, and heptapeptide domain 2. The SARS-CoV-2 Omicron subvariant BA.2.86 exhibits numerous (34) amino acid changes in its spike protein compared to its ancestral strain BA.2. The spike protein mutations in BA.2.86 may confer enhanced resistance to neutralization by vaccine-induced antibodies. Additionally, hybrid immunity, resulting from a combination of vaccination and natural infection, may offer rapid protection against variants like BA.2.86 [12]. Previous studies have demonstrated that the spike protein is a pivotal target for the development of effective vaccines compared to other subunit vaccine approaches. In addition, it has the potential to trigger the specific formation of neutralizing antibodies [13]. More importantly, the Fc region, a tail fragment of antibodies, plays a crucial role in activating the immune system by genetically fusing the Fc domain to antigens. In addition, Fc fusion technology provides a valuable tool to improve the stability and solubility of the protein partner [14].

Conventional vaccines (CVs) can be developed with live attenuated viruses, proteins or inactivated pathogens and are a proven method to induce immune responses against infectious diseases. CVs remain an unsuitable tool, while some pandemic diseases pose a challenge due to unknown pathogens or the time required to develop vaccines. In addition, mutant strains can evade the protective mechanism of CVs [15]. Hence, numerous advanced vaccines have been developed to combat the pandemic situation. Viral vector vaccines (VVVs) often exhibit superior immunogenicity compared to conventional subunit vaccines, eliciting potent humoral and cellular responses that are crucial for eliminating pathogens. Moreover, VVVs can induce stable immunity after a single dose [16]. VVVs have demonstrated efficacy in preclinical and clinical vaccine trials against various pathogens, including HIV, *Plasmodium* spp., Ebolavirus, and SARS-CoV-2, highlighting their versatility in infectious disease prevention strategies [17].

Adenoviruses (AdVs) are a promising area of research for vaccine development, particularly because of their potential speed and ability to elicit broad immune responses. AdVs are non-enveloped viruses with a linear, ds-DNA genome (34–43 kb) that can be easily manipulated. Adenoviral vectors have been developed for various non-communicable diseases, including cardiovascular diseases, cancer, and other serious diseases such as metabolic syndrome, muscle diseases, immunodeficiency syndrome, and neurological disorders [18]. In addition, AdVs have the potential for protein expression and high intracellular gene transfer efficiency, making them an excellent vaccine carrier for infectious diseases [19]. Adenoviral vaccines have been used extensively in the development of novel genetically engineered vaccines. Adenoviral vaccines have several advantages, including rapid production, high safety, and superior efficacy in gene transfer with robust humoral and cell-mediated immune responses [20]. Therefore, adenoviral vaccines are considered a viable approach for the development of new viral vaccines.

The Fc has emerged as a pivotal component in contemporary vaccine development owing to its numerous advantages, including quick purification, extended half-life, and the enhanced immunogenicity of target antigens [21]. It has consistently been shown in previous studies that Fc-fused protein vaccines for H5N1 [22], SARS-CoV-1 [23], and MERS-CoV [24] possess a higher immunogenicity than their Fc-deficient counterparts. In immunotherapy and vaccination, antibodies with Fc regions recruit immune defenses through binding to Fc receptors and eliciting diverse immune responses. Through complement-mediated lysis, phagocytosis, and immune cell recruitment, Fc activities play a major role in neutralizing viruses and virus-infected cells. As a result of Fc-mediated internalization, Toll-like receptors are activated, as well as phagocytosis by T cells through enhanced antigen-presenting cells. Vaccines with neutralizing functions can provide broad protection against multiple pathogens and potentially improve vaccine efficacy. It appears that the Fc fusion approach has the potential to enhance the immunogenicity of SARS-CoV-2 vaccines [25]. A new approach was investigated in this study, where we created an adenovirus vector expressing a human Fc domain along with the SARS-CoV-2-RBM. As part of our evaluation of the RBM_hFc-expressing AdV’s potential as a vaccine candidate, we conducted immunological tests to determine its immunogenetic properties.

## 2. Materials and Methods

### 2.1. Cell Culture

HEK293, A549 and Huh7 cells were obtained from the Korean Cell Line Bank (KCLB: Seoul, Republic of Korea). HEK293 and Huh7 cells were cultured in Dulbecco Modified Eagle Medium (DMEM: Biowest, Nuaillé, France). A549 cells were cultured in Roswell Park Memorial Institute Medium (RPMI, Biowest, France). All restriction enzymes (*Pac*I, *Bam*HI, and *Hin*dIII) were purchased from NEW England BioLabs, Ipswich, MA, USA. All cells were inoculated with 10% fetal bovine serum (FBS) (Gibco: New York, NY, USA) and antibiotics (Biowest: France), and were incubated at 37 °C, 5% CO_2_.

### 2.2. Construction of Recombinant Adenovirus

The receptor binding motif (RBM), which consists of 72 amino acids, was amplified from the SARS-CoV-2 spike gene, which was codon-optimized and commercially synthesized by Genwiz (Suzhou, China). The sequences of the primers for RBM were as follows, (s) 5′-CCG CTC GAG ATG AAT TCT AAC AAT CTT GAT TCT AAG G-3′, (as) 5′-GC TCT AGA TCA TTT ACC CGG AGA CAG GGA GAG GC-3′. Amplification was performed in a 20 µL PCR mixture using the MiniAmp Thermal Cycler (Thermo Fisher Scientific: Walmart, MA, USA). The following programs were recommended for amplification of the target PCR product: denaturation at 94 °C for 5 min, denaturation at 94 °C for 30 s (32 cycles), annealing at 58 °C for 30 s, extension at 72 °C for 45 s, and a final extension at 72 °C for 10 min. The amplified PCR product was extracted using a gel purification kit (Gel Sv) (Geneall Biotechnology Co., Ltd.: Seoul, Republic of Korea) and injected into the *Bam*HI (#R0136S) and *Hin*dIII (#R0104S) site of the pacAd5CMV-N-pA shuttle vector (Cell Biolabs Inc.: San Diego, CA, USA). In addition, human Fc (hFc) was isolated from the pINFUSE-hIgG1-Fc1 vector (InvivoGen: San Diego, CA, USA) and a Myc tag was inserted between the RBM and hFc domains. Finally, the clone RBM_hFc has been constructed into the pacAd5CMV-N-pA vector.

The RAPAD CMV Adenoviral Expression System (Cell Biolabs Inc.: USA) was used to produce recombinant adenoviruses expressing the target gene as described in the manual. In brief, the PacAd5 9.2-100 vector and pacAd5 CMVK-NpA shuttle vector containing RBM_hFc (Ad/RBM_hFc) were linearized with *Pac*I (New England BioLabs: USA; #R0547S) for 6 h at 37 °C. The linearized vectors were purified using DNA purification kit (QIAGEN: Hilden, Germany), then co-transfected into HEK293 cells (1 × 10^6^ cells per 60 mm culture dish), resulting in the generation of recombinant viruses expressing the target gene (RBM_hFc). Cytopathic effect (CPE) was observed with clear plagues in HEK293 cells 10 days post-transfection. After observation of plaque formation, the crude viral lysate was recovered from the cells by three freeze–thaw cycles. Finally, the viral supernatant was aliquoted and stored at −80 °C for further processing after centrifugation at 3000× *g* for 10 min to remove cell debris. A control virus was generated by a similar method using the pacAd5 CMV-GFP control vector.

### 2.3. Overexpression of RBM_hFc in Adenoviral Vector

#### 2.3.1. Western Blotting

Western blotting was performed to analyze the expression of the SARS-CoV-2 RBM_hFc protein. Samples were separated by 10% sodium dodecyl sulfate-polyacrylamide gel electrophoresis (SDS-PAGE) and transferred to a PVDF membrane at 250 mA for 2 h. The membrane was then blocked with 5% skim milk for 1 h to prevent non-specific binding. The Myc-tag antibody (dilution 1:5000, Abcam: Cambridge, UK) was incubated with the membrane overnight at 4 °C. After washing the membrane with 0.1% TBST, an HRP-conjugated secondary antibody was added and incubated for 2 h at room temperature. Finally, an enhanced chemiluminescence solution was used to detect the bands.

#### 2.3.2. Immunofluorescence Assay

Vero cells were seeded in 12-well plates at a density of 1 × 10^5^ cells/well. The cells were then transfected with 1.5 µg of pAdS/RBM_hFc DNA per well. After transfection, the cells were incubated at 37 °C for 48 h under 5% CO_2_ condition. After incubation, the culture medium was removed from the wells. The cells were fixed with 4% paraformaldehyde solution (GeneAll Biotechnology Co., Ltd.: Seoul, Republic of Korea) for 15 min at RT. After the cells were washed three times with PBS, permeabilization was performed with PBS-T (PBS with 0.1% Triton X-100) for 15 min at room temperature. Cells were again washed three times with PBS and blocked with 3% BSA in PBS for 1 h at RT on a shaking platform. The cells were incubated overnight at 4 °C with the Myc antibody (dilution 1:500, Abcam: Cambridge, UK) in blocking solution. The cells were washed three times with PBS-T (PBS with Tween-20). Finally, the cells were incubated for 1 h with an anti-rabbit IgG antibody conjugated with Alexa Fluor® 488 (dilution 1:1000, Abcam: Cambridge, UK).

### 2.4. Amplification of Adenovirus

HEK293 cells were seeded one day prior to infection in a 100-mm dish at a density of 1 × 10^5^ cells/well. To enhance virus attachment, cells were infected with a 1:1 mixture of virus and serum-free medium and incubated overnight at 37 °C and 5% CO_2_. After incubation, the inoculum was removed and replaced with fresh medium containing 5% FBS and antibiotics. The cells were monitored for cytopathic effects (CPE) under incubation conditions of 37 °C and 5% CO_2_. The supernatant was collected by centrifugation at 1500× *g* for 10 min at 4 °C. Finally, the cell monolayer was washed with PBS and lysed by performing three cycles of freezing and thawing. Cell lysates were collected by centrifugation at 1500× *g* for 10 min at 4 °C. The harvested viruses were then propagated in fresh HEK293 cells and purified by CsCl density gradient centrifugation. Finally, they were resuspended in storage buffer (10 mM Tris-HCl (pH 8.0), 4% sucrose, and 2 mM MgCl_2_) and stored at −80 °C.

### 2.5. Quantitative Analysis of Adenoviruses by Crystal Violet Approach

HEK293 cells were seeded on 96-well plates at a concentration of 1 × 10^4^ cells/well. Pure adenoviruses were serially diluted 10-fold from 10^−1^ to 10^−10^ in media containing 5% FBS and antibiotics. The serially diluted viruses were seeded on HEK293 cells and incubated at 37 °C and 5% CO_2_ in an incubator. When CPE was confirmed, the supernatant was removed and 100 µL of crystal violet was aliquoted each well. The cells were stained, washed, the CPE was visually checked and then the titer was calculated.

### 2.6. Confirmation of AdV by PCR and Western Blotting

To determine the expression of the RBM_hFc target protein after adenoviral administration, we performed PCR and Western blot analyses. Viral DNA was extracted from the supernatant of the AdV culture using the Ribospin vRD kit (Geneall Biotechnology Co., Ltd.: Seoul, Republic of Korea). The extracted viral DNA and the purified recombinant plasmid served as templates for PCR amplification with primers specific for the RBM region of SARS-CoV-2 and the human *AdV IX* gene.

The recombinant AdV was used to infect human lung epithelial (A549) and human hepatoma (Huh7) cell lines at a multiplicity of infection (MOI) of 1. Whole cell lysates were harvested 72 h post-infection. Western blot analysis was then performed. Lysates were denatured by boiling in 5× sample buffer at 99 °C for 10 min and separated on an 8% SDS-polyacrylamide gel. The proteins were transferred to a PVDF membrane and blocked with 5% skim milk in TBST for 1 h at RT. Immunodetection was performed with a 1:5000 diluted anti-human IgG-HRP-Fc antibody (Cusabio: Houston, TX, USA; #PA00540F0Rb). Specific protein bands were visualized using a chemiluminescent detection reagent.

### 2.7. Immunization of Mice

Six-week-old female BALB/c mice (Samtako Co., Ltd.: Seoul, Republic of Korea) were randomly divided into two groups. The experimental group received a single intramuscular injection of RBM_Fc AdV, while the control group was injected with an AdV expressing EGFP. Serum samples were collected via retro-orbital plexus puncture at designated time points. Animals were euthanized by cardiac puncture at the end of the study.

### 2.8. Antibody Assay

#### 2.8.1. Enzyme-Linked Immunosorbent Assay

An enzyme-linked immunosorbent assay (ELISA) was developed for the detection of anti-SARS-CoV-2 antibodies. RBM_hFc protein purified with a c-Myc-tagged protein mild purification kit (MBL International: Schaumburg, IL, USA; #011FA) was applied to ELISA plates and incubated overnight at 4 °C. After washing with phosphate-buffered saline with Tween-20 (PBS-T), the plates were blocked with 3% skim milk in PBS for 1 h at RT. The mouse serum was diluted in the blocking solution (1:1000) and incubated overnight at 4 °C. After five washes with PBS-T, horseradish peroxidase (HRP)-conjugated anti-mouse IgG was added and incubated for one hour at RT. After a final PBS-T wash, a tetramethylbenzidine (TMB) substrate solution (Koma Biotech: Seoul, Republic of Korea) was added for color development. The reaction was stopped with sulfuric acid (Koma Biotech: Seoul, Republic of Korea), and the absorbance was measured at 450 nm using a spectrophotometer.

#### 2.8.2. Enzyme-Linked Immunospot Assay

Cytokine secretion by peripheral blood mononuclear cells (PBMC) in response to AdV/RBM_hFc vaccine stimulation was quantified using enzyme-linked immunospot (ELISpot) assay as previously described [26] with minor modification. Immunized mice were euthanized by cardiac puncture, and spleens were harvested. Spleens were mechanically disrupted into a single-cell suspension using a cell strainer (SPL Life Sciences Co., Ltd.: Seoul, Republic of Korea) within a 50 mL conical tube. Red blood cells were lysed, and splenocytes were filtered and cultured with purified RBM_hFc as a stimulatory protein for 48 h at 37 °C. Following cell counting, splenocytes were seeded at a density of 1 × 10^5^ cells/well in a 96-well plate for ELISpot analysis. After 15 h, plates were washed with PBS-T and incubated with a 100 µL diluted detection antibody for 1.5 h at RT. Subsequently, plates were washed and incubated with streptavidin-alkaline phosphatase conjugate for 1 h at RT. After washing with distilled water, BCIP/NBT substrate was added to visualize spot formation. Plates were washed with distilled water and dried overnight. The number of cytokine-producing cells was quantified by counting spots using an ELISpot reader.

### 2.9. Statistical Analysis

All experiments were performed with three replicates and analyzed with SPSS version 16.0 (SPSS Inc., Chicago, IL, USA) using an independent samples *t*-test. The means ± standard deviations of the data were displayed as * *p* < 0.05, ** *p* < 0.01, *** *p* < 0.001, and **** *p* < 0.0001.

## 3. Results

### 3.1. Construction of Recombinant Replication-Defective AdV Expressing RBM_hFc

**Figure 1:**
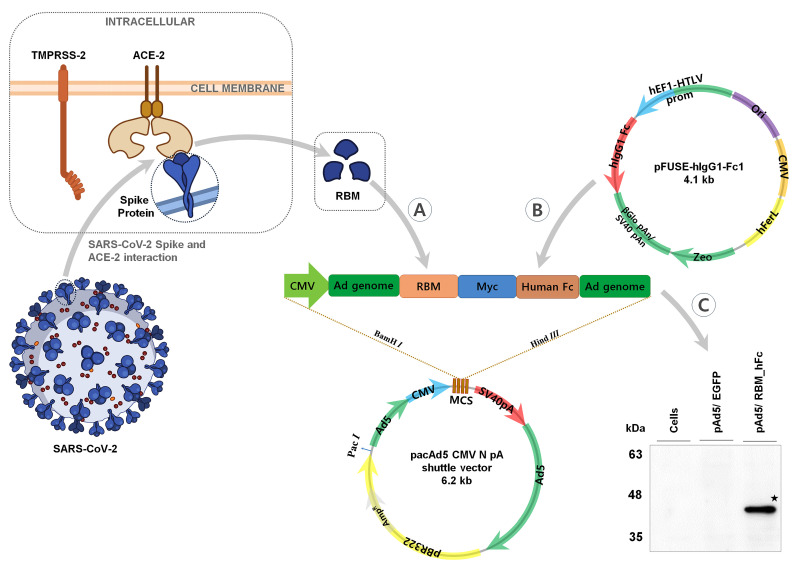
Schematic representation of RBM_hFc construct and protein expression. (**A**): Schematic of the SARS-CoV-2 receptor binding motif (RBM) amplified from the spike protein. (**B**): Schematic of the human Fc domain derived from the pINFUSE-hIgG1-Fc1 vector. (**C**): Schematic of the RBM_hFc fusion protein cloned into the pacAd5-CMV-N-pA vector. Western blot analysis confirms expression of the RBM_hFc fusion protein (★) in HEK293 cells using anti-Myc antibodies. ★ indicates the position of the protein expression.

The RBM of the SARS-CoV-2 spike protein was successfully amplified and cloned into the pAd5 vector, resulting in a fusion protein with the human Fc (hFc) domain, which is derived from the pINFUSE-hIgG1-Fc1 vector (Figure 1A,B). The construct was sequence-verified (Cosmogenetech Co., Ltd.: Seoul, Republic of Korea).

Transient transfection of HEK293 cells with the pAd5/RBM_hFc plasmid led to the expression of a recombinant RBM_hFc protein as confirmed by Western blot analysis using anti-Myc antibodies (Figure 1C). The protein exhibited an apparent molecular weight of approximately 40 kDa on a polyvinylidene difluoride (PVDF) membrane, consistent with the predicted molecular weight of the RBM, Myc tag, and hFc domain.

**Figure 2:**
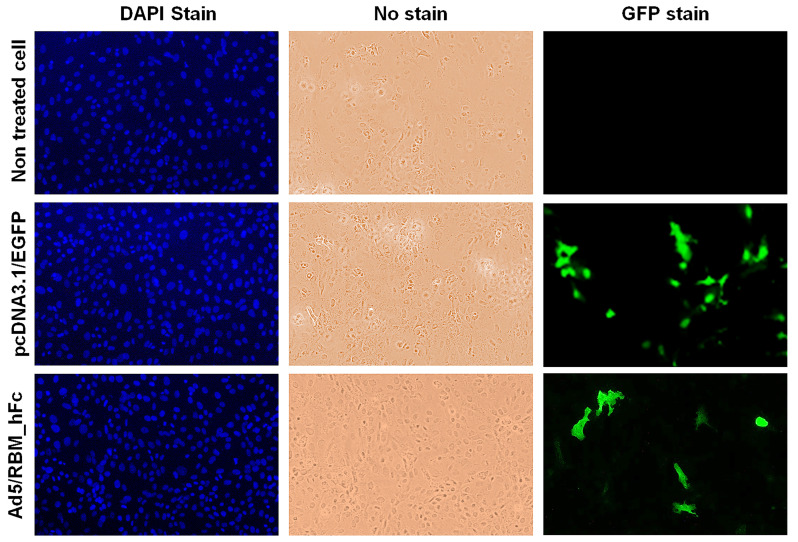
Immunofluorescent detection of RBM_hFc expression in HEK293 cells. HEK293 cells were transfected with Ad5/RBM_hFc or a control EGFP-expressing vector. Cells were fixed, permeabilized, and stained with anti-rabbit IgG Alexa Fluor 488 secondary antibody. Nuclei were counterstained with DAPI. EGFP expressing vector was used for assay control. All samples were examined microscopically at 40× magnification.

To confirm RBM_hFc expression in HEK293 cells, immunofluorescence staining was performed. Cells were fixed, permeabilized, and incubated with an anti-rabbit IgG Alexa Fluor 488 secondary antibody. Nuclei were counterstained with DAPI stain. An EGFP-expressing vector served as a positive control (Figure 2). Minimal background fluorescence was observed in uninfected cells, while DAPI staining revealed intact nuclei. Green fluorescence, indicative of EGFP expression, was detected in control cells.

### 3.2. Generation of Recombinant AdV Expressing SARS-CoV-2 RBM Gene

**Figure 3:**
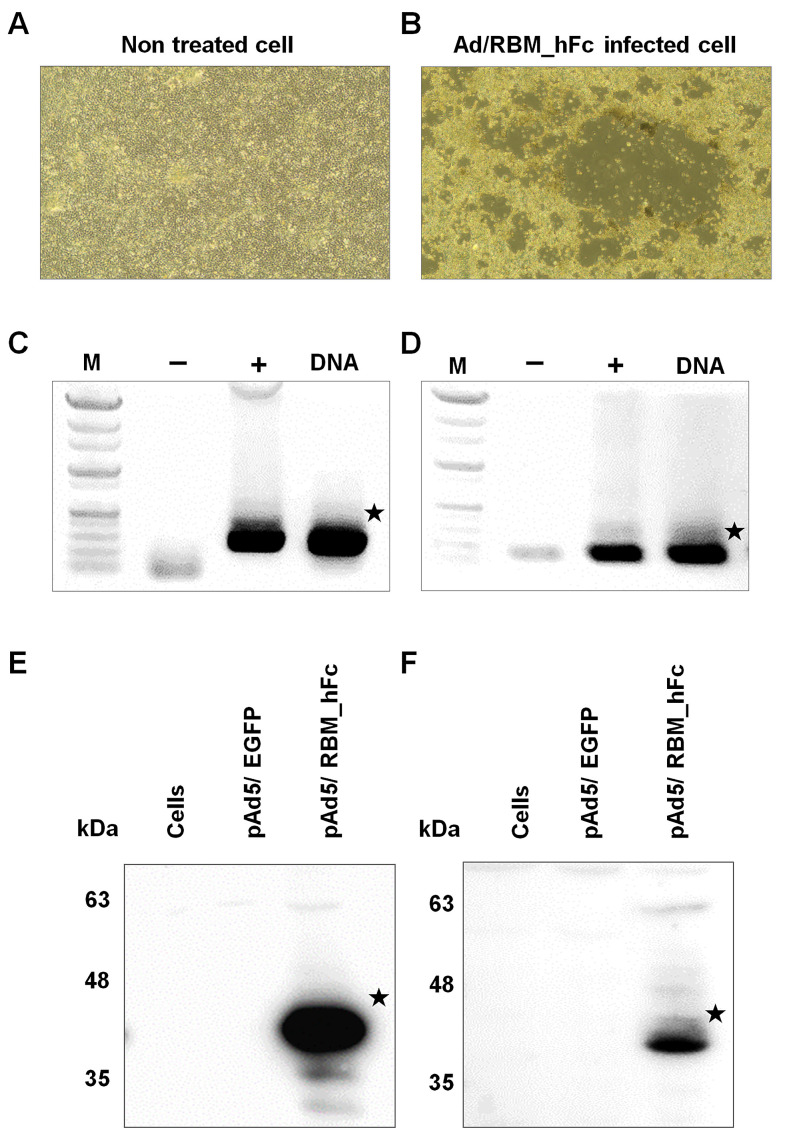
Generation and characterization of pAd5/RBM_hFc adenovirus. (**A**): Non-treated HEK293 cells were considered as a control. (**B**): Cytopathic effect (CPE) observed in HEK293 cells 10 days post-transfection with the Ad/RBM_hFc adenoviral vector. All samples were examined microscopically at 40× magnification. PCR analysis confirming the presence of adenoviral DNA and RBM gene. Viral DNA extracted from harvested virus was used as template for amplification of the RBM gene (201 bp) (**C**) and adenoviral IX gene (120 bp) (**D**), M (marker)—1 kb DNA ladder (Biofact). Western blot analysis of cell lysates from A549 (**E**) and Huh7 (**F**) cells transduced with pAd5/RBM_hFc. Protein expression was detected at around 40 kDa using an anti-human Fc antibody. A plasmid containing the EGFP served as a negative control. ★ indicates the position of the target gene amplification (**C**,**D**) and protein expression (**E**,**F**).

Both PacAd5 9.2-100 vector and Ad/RBM_hFc vector were linearized by *Pac*I restriction enzyme digestion for 6 h at 37 °C. Replicable AdV expressing the target protein was generated through co-transfection of these linearized vectors into HEK293 cells, an E1-complementing cell line. The emergence of viral plaques was observed on day 10 post-transfection compared with control (Figure 3A,B). Viral titer was subsequently amplified through three to four rounds of viral propagation by inoculating fresh HEK293 cells with harvested viral supernatant.

To confirm viral presence, PCR analysis was performed on DNA extracted from the harvested virus. Primers specific to the RBM were used to amplify the target gene and confirmed the size as 201 bp (Figure 3C). Additionally, PCR targeting the *AdV IX* gene, a capsid protein, was conducted to verify adenoviral genome integrity at the size of 120 bp (Figure 3D).

The AdV expressing RBM_hFc was transduced into A549 and Huh7 cells. Cell lysates were harvested and subjected to SDS-PAGE followed by Western blotting onto a PVDF membrane. Immunodetection using a human Fc-HRP conjugated antibody revealed a band at approximately 40 kDa in both cell lines, indicating the presence of RBM_hFc protein. No corresponding band was observed in cells transduced with the control Ad5/EGFP virus (Figure 3E,F).

### 3.3. Humoral Immune Response Induced by AdV-Delivered RBM_hFc Vaccine in Mice

**Figure 4:**
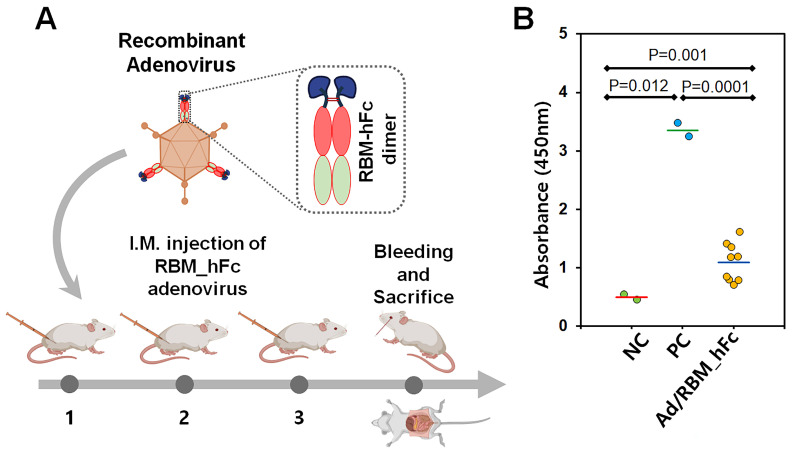
Humoral immune response to Ad/RBM_hFc vaccination. (**A**): BALB/c mice received intramuscular injections of Ad/RBM_hFc or control vaccine at two-week intervals. (**B**): Serum IgG titers against RBM were determined by ELISA. Mice immunized with Ad/RBM_hFc exhibited significantly higher RBM-specific IgG levels compared to the control group. Data are presented as mean ± standard deviation from three independent experiments. The bars represent mean value.

To evaluate the immunogenicity of the adenoviral vaccine encoding SARS-CoV-2 RBM_hFc, groups of five BALB/c mice were immunized intramuscularly at two-week intervals (Figure 4A). Serum samples were collected via cardiac puncture and subjected to centrifugation. IgG titers were determined by enzyme-linked immunosorbent assay (ELISA) using RBM_hFc as the coating antigen. Ad/EGFP-immunized mice served as a negative control. Mice immunized with SARS-CoV-2 spike (positive control) exhibited significantly higher IgG titers (mean (M)—3.353 at 450 nm; standard deviation (SD)—±0.162; and 95% confidence interval (95% CI)—1.455 [1.899 to 4.808]) compared to the negative control group (M—0.494 at 450 nm; SD—±0.062; and 95% CI—0.559 [−0.065 to 1.053]; *p* = 0.012). Mice immunized with Ad/RBM_hFc exhibited significantly higher IgG titers (M—1.090 at 450 nm; SD—±0.326; and 95% CI—0.250 [0.839 to 1.340]) compared to the negative control (*p* = 0.001) and positive control (*p* = 0.0001) groups (Figure 4B). These results demonstrated that AdV/RBM_hFc significantly induced IgG-mediated humoral immune responses.

**Figure 5:**
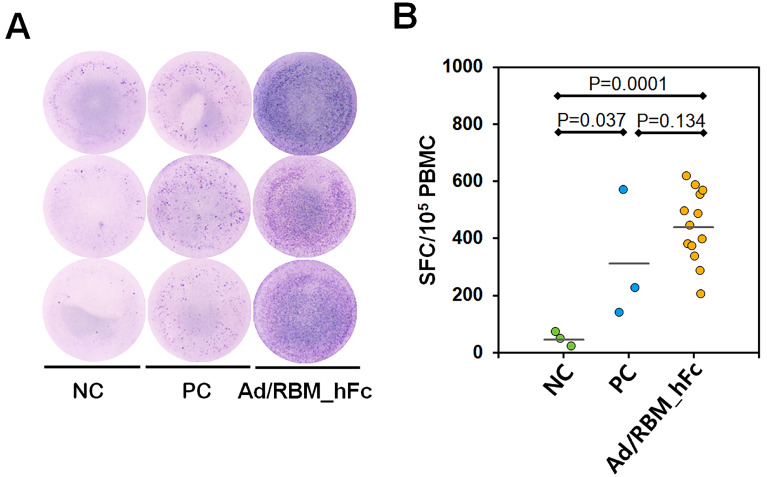
Immune response in splenocyte cells towards AdV/RBM_hFc vaccination. (**A**): Cytokine spot-forming cells were highlighted by ELISpot Immunoassay. Representative ELISpot images showing cytokine-secreting cells as individual spots. (**B**): Quantification of spot-forming cells in splenocytes by ELISpot reader. NC: negative control (Ad/EGFP induced splenocyte cells); PC: positive control (SARS-CoV-2—S induced splenocyte cells); Ad/RBM-hFc: SARS-CoV-2—RBM_hFc induced splenocyte cells. Data are presented as mean ± standard deviation from three independent experiments. The bars represent mean value.

To assess the cellular immune response, an ELISpot assay was performed to quantify cytokine-producing cells. ELISpot assay results revealed a significantly increased number of cytokine-secreting cells in splenocytes from AdV/RBM_hFc-immunized mice compared to the non-immunized mice. In brief, splenocytes from SARS-CoV-Spike immunized mice have been used as a positive control, secreting high number of cytokines (M—310.667 spot-forming cells (SFC); SD—±227.913; and 95% CI—566.167 [−255.501 to 876.834]) compared to negative control (Ad/EGFP immunized mice) (M—46 SFC; SD—±25.515; and 95% CI—63.382 [−17.382 to 109.382]; *p* = 0.037). While splenocytes from AdV/RBM_hFc-immunized mice yielded maximum cytokine spots (M—440 SFC; SD—±124.976; and 95% CI—75.522 [364.478 to 515.522]) compared to the negative control (*p* = 0.0001) and positive control (*p* = 0.134) (Figure 5). These results demonstrated that AdV/RBM_hFc significantly induced cytokine responses compared to natural spike protein of SARS-CoV-2.

## 4. Discussion

In late 2019, SARS-CoV-2, a highly contagious and pathogenic coronavirus, was first detected. It is an extremely dangerous respiratory disease that poses a serious threat to public health and safety [27]. Spike protein initiates the infection process through viral spike interaction with host receptors, which is critical for SARS-CoV-2 entry into host cells. Due to SARS-CoV-2, significant investment was made in the development of a COVID-19 vaccine targeting S protein [28]. A cryogenic electron microscopy study of the RBD model indicated that the receptor-binding motif (RBM) directly interacts with ACE2, which acts as an entry point for SARS-CoV-2 [29]. In addition, RBM of SARS-CoV-2 emerged as a promising antigen for subunit vaccine development due to its potential to elicit a robust neutralizing antibody (nAbs) response [30]. Researchers have demonstrated that nAbs (C144 and REGN10933) specific for RBD prevent virus entry by inhibiting spike protein interactions with ACE2 receptors [8,31]. The Fc region of antibodies plays a crucial role in initiating immune responses against target pathogens. Consequently, researchers are incorporating Fc domains into vaccine design to enhance immunogenicity against various infectious diseases compared to vaccines without Fc domains [32]. To address the pandemic, the development of a safe and efficacious vaccine capable of eliciting robust, durable virus-specific immune responses is essential [33]. Therefore, in our study, we constructed a recombinant adenovirus (AdV) expressing both the RBM and human Fc (hFc).

Initially, we constructed a pacAd5CMV-N-pA shuttle vector containing the RBM (201 bp), hFc of IgG, a CMV promoter, and a Myc tag. After successful cloning, protein expression was confirmed by Western blot analysis. Results demonstrated the expression of the fusion protein at approximately 40 kDa, consistent with the predicted molecular weights of the RBD, Myc tag, and hFc. This type of construction offers several advantages, including high transgene expression in mammalian cells, rapid detection or purification of the target protein, and the ability to elicit strong gene-specific antibody responses [34]. Immunofluorescence assay confirmed the expression of the fusion protein in mammalian cells. Results indicated that the transfection efficiency of the construct into 293T cells was outrageous. As in previous studies [35], once the stable cell line was confirmed and infected with AdV, CPE was characterized by degenerative morphological changes associated with viral replication and were observed at 48 h post-infection in HEK293 cells. The cells exhibited rounding and detachment over time. Our results suggest that infection with AdV/RBM_hFc induces pronounced CPEs within a short period (48 h), compared to recent studies that reported noticeable CPEs at 96 h [36]. It proved that AdV/RBM_hFc was more efficacious than pBRSAdVGZ3-12 constructs.

In this study, we evaluated AdV vaccine efficacy and immunogenicity using ELISA and ELISpot on murine sera. Considering our limitation, we did not employ more advanced diagnostic techniques such as PRINT immunoassay, competitive ELISA neutralization assays, or pseudovirion neutralization assays, which could provide more comprehensive insights into vaccine-induced protective immunity. Previous study has demonstrated that fusion proteins comprising hepatitis B viral antigen and Fc domains or synthetic peptides elicited augmented viral-specific CD8^+^, CD4^+^, and B cell responses [32,37]. In another study, ELISA result demonstrated enhanced antibody responses (IgG and IgA) in chickens immunized with avian metapneumovirus (aMPV) containing chicken Fc (cFc) domains [38]. Moreover, chimeric pseudorabies virus incorporating Fc domains induced elevated levels of varies types of immunoglobulins such as, IgG2a, IgG1, and IgG in mice, providing superior protection against lethal challenge compared to the inactivated wild type virus [39]. Similarly, in this study, ELISA data revealed significantly increased IgG levels in mice immunized with AdV/RBM_hFc compared to controls. These results indicate a superior immunogenic response (IgG; ~twofold) induced by the effective expression of RBM and hFc on AdVs.

Cytokine production by dendritic cells in response to viral immune complexes can occur via several mechanisms [40]. One possible pathway is cytosolic recognition of adenovirus–antibody complexes by the intracellular Fc receptor, leading to enhanced cytokine secretion [41]. ELISpot assay can quantify the immune related cells, particularly T cell responses and cytokines against vaccines [42]. This technique has been widely employed to evaluate immune responses in vaccine development for various infectious diseases, including HIV [43], tuberculosis, and malaria [44], facilitating the assessment of prophylactic and therapeutic vaccine candidates. Similarly, aMPV-cFc can elevate inflammatory cytokine production, such as IL-1β, and IFN-γ in chickens [38]. Recent research has demonstrated that IFN-γ was released in significant concentrations following exposure to the Ad26.COV2.S and ChAdOx1 nCoV-19 vaccines, but not in the case of SARS-CoV-2 spike protein peptides, such as SP1 and SP2 [26]. Similarly, our AdV vaccine, which combines RBM and hFc, demonstrated a significant increase in the number of cells secreting specific cytokines (IFN-γ) and analyzed the distribution of cytokine-producing cells and their relative frequencies after 48 h. These results suggest that AdV/RBM_hFc may be a promising candidate for enhancing immunogenicity against SARS-CoV-2.

Adenoviral vectors have a proven safety record and are used therapeutically in clinical settings [45]. They have the capacity to elicit both humoral and cellular-mediated immune responses following a single immunization. This makes them well-suited for emergency preventive measures during pandemics [46]. Therefore, several effective adenoviral vaccines encoding the SARS-CoV-2 S protein were developed using adenoviral vectors within a short period of time (2020–2022). For example, the recombinant adenovirus type 5-vectored vaccine encoding COVID-19 spike protein (Convidecia vaccine—CanSino Biologics: Tianjin, China) [47], the chimpanzee adenovirus vectored vaccine encoding the SARS-CoV-2 spike protein (AstraZeneca vaccine—Oxford University and AstraZeneca: London, UK) [48], the recombinant adenovirus type 26-vectored vaccine encoding full length SARS-CoV-2 spike protein (Ad26.COV2.S vaccine—Janssen Vaccines: Beerse, Belgium) [49,50], and the recombinant adenovirus type 26 (first dose)- and adenovirus type 5 (second dose)-vectored vaccine encoding full length SARS-CoV-2 spike protein (Sputnik V vaccine—Gamaleya: Moscow, Russia) [45] have been successfully deployed to combat the SARS-CoV-2 pandemic. A single immunization with an Ad5-vector based vaccine encoding the SARS-CoV-2-S protein elicited robust humoral and cellular immune responses in most recipients, demonstrating the safety profile of the vaccine [51]. The chimpanzee adenovirus vector vaccine, ChAdOx1, harboring the SARS-CoV-2 S protein, induced S protein-specific T-cell responses on day 14, IgG responses on day 28, and neutralizing antibodies (91%) in humans [48]. In 2020, Mercado et al. demonstrated that an Ad26 vector expressing the full-length SARS-CoV-2 spike protein can induce robust immunogenicity and efficacy in rhesus macaques [52]. In a human model, the Ad26.RSV.preF vector, which encodes the respiratory syncytial virus (RSV)-fusion protein, has been demonstrated to increase neutralizing antibodies by 5.8-fold and protect humans from RSV infection [53]. Large-scale clinical trials have also demonstrated significant immunogenicity, as evidenced by strong T-cell (CD4^+^ and CD8^+^) and antibody responses following immunization with Ad26.COV2.S [54].

Most of the adenoviral vector vaccines commonly induce transient adverse effects, including fatigue, headache, myalgia, fever, and pain at the injection site [55]. Recent studies have reported rare cases of vaccine-induced immune thrombotic thrombocytopenia following immunization with adenovirus-based vaccines like Ad26.COV2.S and ChAdOx1-S. This disorder is a critical condition characterized by blood clots and low platelet counts, which can lead to bleeding and, in some cases, death [56,57]. In 2023, Aid et al. [58] highlighted that the Ad26.COV2.S vaccination has been associated with transient platelet activation and coagulation, particularly in patients who subsequently developed thrombosis with thrombocytopenia syndrome. Due to safety concerns, some adenovirus-based RSV vaccine candidates, such as Ad.26.RSV.preF, RSVPreF3, RSVpreF, MEDI7510, and mRNA-1345, have been discontinued or paused in clinical trials [59]. To address these issues, extensive research is underway to develop novel adenovirus-based vaccines with unique components (for example, combination of Ad26.RSV.preF and RSV preF protein), optimized dosing regimens, and enhanced stability [59,60]. These vaccines aim to overcome the limitations observed with previous Ad26-based vaccines associated with safety concerns.

According to [61], the pseudovirus have emerged to evaluate the efficiency of antibodies or vaccines in animal models. For example, the pseudovirus neutralization assay quantifies SARS-CoV-2 infection rates by evaluating the ability of SARS-CoV-2 neutralizing antibodies or vaccine-induced immunity to inhibit viral entry into host cells [62]. This versatile approach enables various applications for studying SARS-CoV-2, including vaccine development, therapeutic drug screening, and infection mechanism studies, offering a safe and efficient alternative to live virus experimentation. Similarly, adenoviral vector vaccines demonstrate enhanced immune protection and reduced viral transmission, making them attractive candidates for vaccine delivery. Additionally, in vitro safety studies demonstrated a negligible thrombogenic effect of Ad5-based therapy [63]. Hence, we have developed a novel Ad5-based vaccine expressing RBM fused to human Fc that aims to rapidly induce robust humoral and cellular immune responses. Immunization with Ad/RBM_hFc resulted in significant upregulation of IgG antibodies and cytokines, demonstrating its potential as a SARS-CoV-2 vaccine candidate. Unlike some Ad26-based vaccines that target the full-length spike protein, AdV vaccines specifically target the RBD, a critical region for viral entry. This focused approach may enhance the immune response and protective efficacy. Our study suggests that the Ad/RBM_hFc vaccine can potentially augment both humoral and cellular immunity, making it a promising candidate for future pandemics. However, further preclinical and clinical studies are necessary to fully assess the safety and efficacy of this vaccine approach.

## 5. Conclusions

In this study, we constructed a recombinant adenovirus vaccine candidate expressing the RBD of the SARS-CoV-2 spike protein and hFc on its surface. This Ad5/RBM_hFc vaccine demonstrated a high potential to enhance immunogenicity when delivered via an adenoviral carrier system. The resulting adenovirus elicited robust humoral (two-fold) and cellular (10-fold) immune responses in mice, as accurately assessed by ELISA and ELISpot techniques. These findings suggest that adenovirus-based RBM_hFc vaccines are a safe and promising alternative for COVID-19 prevention. However, further in-depth studies are required to fully evaluate their protective efficacy in preclinical models and ultimately in human clinical trials.

## Data Availability

The data presented in this study are available on request from the corresponding author.
